# Retraction Note: LncRNA BCAR4 promotes migration, invasion, and chemo-resistance by inhibiting miR-644a in breast cancer

**DOI:** 10.1186/s13046-023-02658-5

**Published:** 2023-04-11

**Authors:** Tangwei Wu, Xiaoyi Li, Ge Yan, Zheqiong Tan, Dan Zhao, Shuiyi Liu, Hui Wang, Yuan Xiang, Weiqun Chen, Hongda Lu, Xinghua Liao, Yong Li, Zhongxin Lu

**Affiliations:** 1 Department of Medical Laboratory, The Central Hospital of Wuhan, Tongji Medical College, Huazhong University of Science and Technology, 26 Shengli St., Jiangan District, Wuhan, 430014 China; 2 College of Pharmacy, Hubei University of Chinese Medicine, Wuhan, 430065 China; 3 School of Laboratory Medicine, Hubei University of Chinese Medicine, Wuhan, 430065 China; 4 Cancer Research Institute of Wuhan, The Central Hospital of Wuhan, Tongji Medical College, Huazhong University of Science and Technology, Wuhan, 430014 China; 5 Key Laboratory for Molecular Diagnosis of Hubei Province, The Central Hospital of Wuhan, Tongji Medical College, Huazhong University of Science and Technology, Wuhan, 430014 China; 6 Department of Oncology, The Central Hospital of Wuhan, Tongji Medical College, Huazhong University of Science and Technology, Wuhan, 430014 China; 7 Institute of Biology and Medicine, College of Life and Health Sciences, Wuhan University of Science and Technology, Wuhan, Hubei 430081 People’s Republic of China; 8 Department of Medicine, Dan L Duncan Comprehensive Cancer Center, Baylor College of Medicine, Houston, TX 77030 USA

**Retraction Note: J Exp Clin Cancer Res 42, 14 (2023)**

**https://doi.org/10.1186/s13046-022-02588-8**

The Editor-in-Chief has retracted this article. Concerns were raised regarding a number of figures, specifically:

- Fig. 2B: the si-2# invasion panel appears to partially overlap with si-BCAR4 + miR-644a inhibitor invasion panel in Fig. 4E
- Fig. 4E: the control panels for migration and invasion appear to overlap
- Fig. 4E: the si-BCAR4 panels for migration and invasion appear to overlap
- Fig. 4F: the control panels for migration and invasion appear to overlap
- Fig. 5C: the mimic nc and inhibitor nc panels appear to overlap
- Fig. 5C: the mimic nc and miR-644a inhibitor panels appear to overlap

The Editor-in-Chief therefore no longer has confidence in the results and conclusions of this article.

Zhongxin Lu has stated on behalf of all authors that they agree to this retraction.

